# Genetic analysis of chromosome 20-related posterior polymorphous corneal dystrophy: genetic heterogeneity and exclusion of three candidate genes

**Published:** 2008-01-16

**Authors:** S. Mohsen Hosseini, Sarah Herd, Andrea L. Vincent, Elise Héon

**Affiliations:** 1 Program in Genetics and Genome Biology, The Hospital for Sick Children Research Institute, Toronto, Ontario, Canada; 2 Institute of Medical Science, University of Toronto, Toronto, Ontario, Canada; 3 Department of Ophthalmology and Vision Science, The Hospital for Sick Children and University of Toronto, Toronto, Ontario, Canada; 4 Department of Ophthalmology, The University of Auckland and Greenlane Clinical Centre, Auckland, New Zealand.

## Abstract

**Purpose:**

Posterior polymorphous corneal dystrophy (PPCD) is a genetically heterogeneous autosomal dominant condition which maps to the pericentromeric region of chromosome 20. Mutations in the *VSX1* transcription factor have been reported in patients affected with PPCD, keratoconus, or a combination of both phenotypes. However, no mutation was identified in the coding region of *VSX1* in the family used for the original mapping. To clarify the genetic basis of PPCD1, a thorough analysis was performed on the original PPCD1 family and two other PPCD1-linked families. As part of the analysis, the expression profile, transcript variants, and evolutionary conserved regions of *VSX1*, a key candidate gene within the linkage interval, were characterized.

**Methods:**

Haplotype analysis was performed using highly informative markers on the pericentromeric region of chromosome 20. *VSX1* transcript variants were identified using RT–PCR and characterized by 3′RACE assay. Temporal expression profile of *VSX1* was evaluated using semi-quantitative real-time RT–PCR on human tissues. Evolutionary conserved regions (ECRs) were identified in the vicinity of *VSX1* using publicly available sequence alignments (UCSC and rVista) and sequenced for mutation analysis.

**Results:**

Recombination events were identified that narrow the PPCD1-disease interval from 20 to 16.44 cM. This smaller interval includes the CHED1 locus and a recently described PPCD locus in Czech families. The three strongest candidate genes of the PPCD1-CHED1 overlap region (*RBBP9*, *ZNF133*, *SLC24A3*) did not show any mutations in our PPCD1-linked families. Semi-quantitative real-time RT–PCR detected *VSX1* expression in neonatal human cornea. Six transcript variants of *VSX1* were characterized. Four of the transcript variants spliced to two novel exons downstream of the gene. Mutation analysis of the PPCD1-linked families did not reveal any mutations in the full genomic sequence of *VSX1* (considering all splice variants) or in the six *cis*- regulatory modules predicted in the vicinity of *VSX1* (100 kb).

**Conclusions:**

This is the first documentation of *VSX1* expression in human neonatal cornea. We provide evidence for genetic heterogeneity of chromosome 20-related PPCD and refinement of the original PPCD1 interval. The full genomic sequence of *VSX1* and coding exons of three other candidate genes were excluded from being pathogenic in the original PPCD1 family.

## Introduction

Posterior polymorphous corneal dystrophy (PPCD; OMIM 122000, 609140, and 609141) is an autosomal dominant endothelial corneal dystrophy characterized by vesicular lesions and epithelial-like changes of the corneal endothelium. PPCD shows variable expressivity [1,2] and while most cases are mild; severe cases require corneal transplantation (about 11%) [3]. PPCD is an important indication of penetrating keratoplasty for congenital corneal opacities and a significant risk factor for the development of glaucoma [4,5].

PPCD is genetically heterogeneous with linkage reported to three different loci [6-8] and evidence for further locus heterogeneity [9]. PPCD1 (OMIM 122000) was mapped to a 30 cM pericentromeric region on chromosome 20 (between D20S98 and D20S108) using a large three generation family [6]. This interval includes the 2.7 cM region for autosomal dominant congenital hereditary endothelial dystrophy (CHED1; OMIM 121700) [10], suggesting that the two conditions might be allelic variants.

*VSX1*, a paired-like homeobox transcription factor in the PPCD1 disease interval, is an attractive positional and functional candidate gene. Different groups have reported mutations involving functional domains of *VSX1* in patients affected with PPCD [11,12], keratoconus [11,13], or a combination of both phenotypes [11,14]. However, unresolved issues remain concerning the pathogenic role of *VSX1* in PPCD. This gene was not shown to be expressed in adult human cornea [11,15,16]. Mice with null alleles do not show any corneal abnormalities [17]. In addition, no mutations were detected in the family used for mapping the PPCD1 locus (Z_max_=5.54 at θ=0 for marker D20S45) [6,11].

Failure to detect a pathological sequence variant by mutational analysis of the coding sequence (exons 1–5) of *VSX1*, a key candidate gene, in the original PPCD1-linked families [6,11] prompted our group to characterize expression profile and regulatory elements of this gene to identify novel *VSX1* genomic sequences for analysis. We addressed the controversy over corneal expression of *VSX1* by performing a temporal expression analysis in human samples that showed perinatal expression of this gene in human cornea. To address the genetic basis of disease in PPCD1-linked families, we further investigated the pathogenic role of *VSX1*, considering different possible mutation mechanisms involving previously uncharacterized transcript variants or regulatory elements. Finally, further mapping of the disease interval in PPCD1-linked families, with or without a *VSX1* mutation, suggests genetic heterogeneity at the PPCD1 locus.

## Methods

### Human subjects identification

The project was approved by The Hospital for Sick Children Research Ethics Board and was conducted in accordance with the tenets of the Declaration of Helsinki. All participating patients signed an informed consent. Patients were recruited through the ocular genetics clinic of the Hospital for Sick Children and by international collaborators.

Three PPCD affected families with evidence of linkage to PPCD1 were included in the study. The original mapping study of PPCD1 was conducted on family 1 [6]. Family 2 was also linked to PPCD1 (unpublished data). Family 3 was the first PPCD affected family with a *VSX1* mutation identified (G160D) [11]. No *VSX1* mutation had been identified in families 1 or 2 [11].

### RNA extraction

Human eyes or corneas of different ages (12 weeks of gestation to 64 years) were collected within 24 h postmortem from the Eye Bank of Canada (Ontario Division) or a local abortion clinic and preserved in RNALater^TM^ (Ambion, Austin, TX). After rapid dissection, total RNA was isolated using TRIzol® reagent (Invitrogen, Burlington, Canada). DNA contamination was removed by RNase-free DNase I treatment (Roche, Laval, Canada).

### Real-time semi-quantitative RT–PCR

One μg of total RNA was reverse transcribed (SuperScript^TM^ II First-Strand Synthesis, Invitrogen) using random hexamers. Melanin inhibition of RT–PCR was reversed by adding 20 μg of BSA (New England Biolabs, Ipswich, MA) to the reaction after primary denaturation [18].

Real-time PCR was conducted according to standard guidelines (Brilliant® SYBR® Green qPCR Master Mix, Stratagene, La Jolla, CA; Abi Prism® 7500-HT, Applied Biosystems, Foster City, CA). Dissociation curve analysis of amplification products was performed to confirm presence of a single product. To maintain consistency, baseline and threshold values were set automatically by the data analysis software (SDS v2.1, Applied Biosystems). To standardize the amount of cDNA in each reaction, an endogenous reference gene was used as internal control. Relative expressions were calculated based on the C_t_ values according to models previously described [19].

### Rapid amplification of cDNA ends (RACE)

3′ RACE was performed on 1 μg of total retinal RNA (extracted from adult human retina) using FirstChoice® RLM-RACE kit (Ambion) according to standard guidelines [20,21]. Primers used are shown in Table 1 (PCR conditions are available upon request). Final PCR products were electrophoresed on 2% sieving agarose gels. All major visible DNA bands were gel purified (MinElute® or QIAquick Gel Extraction, Qiagen, Mississauga, Canada), ligated into a T-vector (pGEM® T-Easy Vector, Promega, Madison, WI) and transformed into Competent E.coli (Subcloning Efficiency^TM^ DH5α^TM^, Invitrogen). Plasmid DNA was extracted from positive clones (QIAprep® Miniprep, Qiagen). The presence of expected inserts was confirmed by enzymatic digestion (*Eco*RI or *Eag*I, New England Biolabs) before bidirectional sequencing using T7 and SP6 primers. Sequence files for each clone were then assembled into a single FASTA file (CAP3 software provided in the public domain by PBIL) [22] and aligned against the genomic sequence (NT_011387) using the mRNA-to-genomic alignment program Spidey (provided in the public domain by NCBI) [23].

**Table 1:** Sequence of oligonucleotide primers.

**Category**	**Primer Name**	**Sequence**
3′RACE	Outer g.s.* Primer A (3OA)	ATGAGGACAGCCAGTCTGAA
	Inner g.s. Primer A (3IA)	ACCTTGGGCAAGAGGAAGAA
	Outer g.s. Primer B (3OB)	ATGGCCGAGTACGGGCTGT
	Inner g.s. Primer B (3IB)	CAGACTCCGTGCTCAACTCC

Realtime RT–PCR	GAPDH fwd	CAGGGATGATGTTCTGGAGAG
	GAPDH rev	CTGCACCACCAACTGCTTAG
	VSX1 fwd	GGGCAGATAATATACTCCACAAAG
	VSX1 rev	CATTTCTCGGGCATACACATC

VSX1 regulatory elements	R1-R2 fwd	GCCCTGCAAAGTGGGTCT
	R1-R2 rev	CTCACAGCAGGTCCAACCTC
	R3–1 fwd	TGCTAAAGAGCCGCAGATTG
	R3–1 rev	GATGCACTTGTCTCCTCGTG
	R3–2 fwd	CTCTTCCCAACTGAAAAATGC
	R3–2 rev	TCTGAAGGAGAGTATTTGATTTCC
	R4 fwd	GAAGCACAAGACAGGGAAGG
	R4 rev	AAGCTGAGGATGTGTTCCTG
	R5 fwd	ACCCAAAACGTCAGGACTTC
	R5 rev	AGCTGTGTTCTTCCCATGC
	R6 fwd	ATGGGCAGGATACTGTGGAG
	R6 rev	TGGAAGGAACTTTGGACCTC

Sequences of other primers will be available upon request. *g.s.: gene specific.

### Whole genome amplification

Genomic DNA was extracted from peripheral blood or buccal swabs [24,25]. In the case of index families, DNA was amplified before application in downstream assays using a ϕ29 polymerase based system (GenomiPhi^TM^, Amersham Biosciences, Piscataway, NJ) [26]. Whole genome amplification methods based on multiple displacement provide a balanced representation of genome suitable for genotyping and sequencing [27,28].

### DNA sequencing

DNA fragments of interest were PCR-amplified [21,29] and purified (QIAquick PCR Purification, Qiagen) before cycle sequencing and sequence analysis as described elsewhere [30].

### Short tandem repeat polymorphism (STRP) genotyping

DNA (20–50 ng) from selected individuals were PCR-amplified, denatured, electrophoresed and detected on an automated sequencer (ALF^TM^, Pharmacia Biotech AB, Uppsala, Sweden). Genotypes were determined by software analysis (Fragment Manager v1.1, Pharmacia LKB Biotechnology, Uppsala, Sweden).

## Results

### Temporal expression profile of human *VSX1*

**Figure 1:**
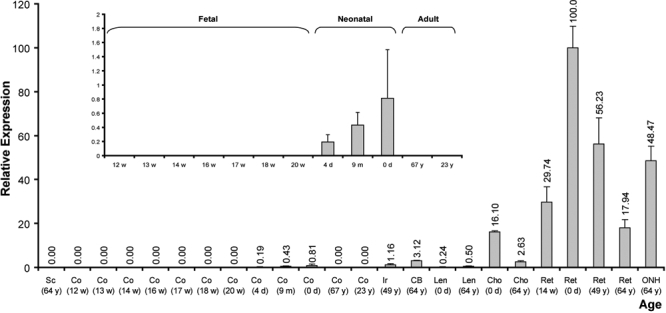
Relative expression of *VSX1* in a panel of eye tissues of differnet ages. Each bar represents the relative expression of *VSX1* normalized to *GAPDH* in a different tissue/age; mean±SD (Sc: sclera, Co: cornea, Ir: iris, CB: ciliary body, Len: lens, Cho: choroid, Ret: retina, ONH: optic nerve head). Numbers above each bar are the average level of expression relative to neonatal retina obtained from triplicates of the experiment. Numbers on the x-axis are the age of donor individuals (w: fetal week, y: year, m: month, d: day). Data for corneal samples is shown expanded in the inset.

To elucidate the temporal expression profile of *VSX1* in the cornea, semi-quantitative RT–PCR was performed on a panel of human eye tissues (adult, neonatal, and fetal). To select the least variable internal control gene, a validation strategy based on the geometric average of multiple control genes was used [31]. Of the housekeeping genes tested (*ACTB*, *GAPDH,* and *Cyclophilin A*), *GAPDH* was selected as the reference gene since it showed the lowest variation (geometric average of 1.401). The experiment was repeated three times in triplicate with each cDNA sample. Figure 1 shows the expression profile of *VSX1* based on relative comparative fold to the housekeeping gene. Real-time PCR on human ocular tissues confirmed expression of *VSX1* in neonatal but not in adult cornea. The highest expression was in retina and in the neonatal period. Lens and neonatal cornea show the lowest levels of expression. No expression was detected in the sclera or in the adult and early fetal cornea (less than or equal to 20 weeks).

### Characterizing *VSX1* transcript variants

EST alignments available in the UCSC genome browser and exon predictions based on EST clustering, such as ECgene v1.2 and AceView, provided evidence of novel exons downstream to *VSX1*. This was confirmed by RT–PCR on human adult retinal RNA using primers in predicted exons that produced PCR products of expected sizes.

**Figure 2:**
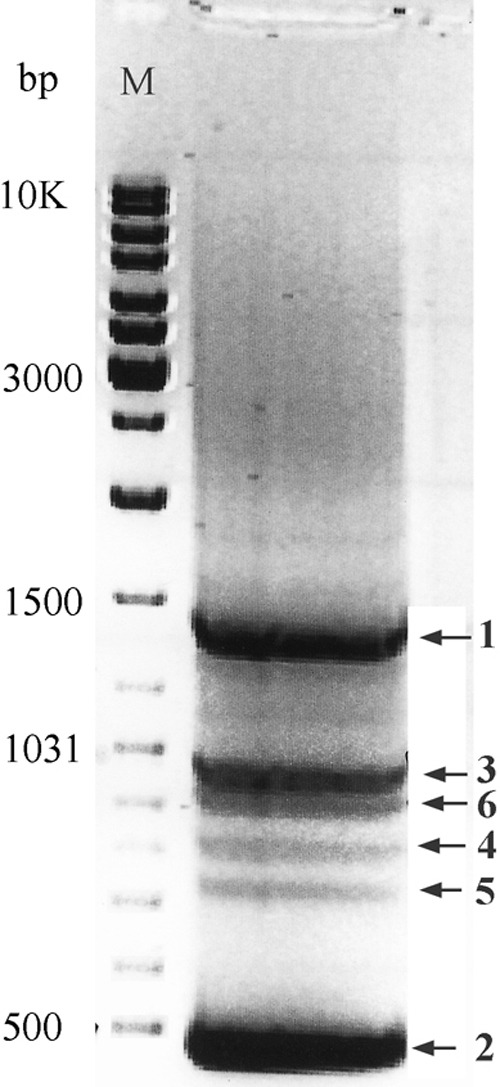
Photograph of ethidium bromide stained 3′RACE products electrophoresed on a 2% sieving agarose gel. Numbers on the left show the sizes of molecular marker (M) bands in bp. Numbers on the right correspond to different *VSX1* transcript variants.

To identify different mRNA classes transcribed from the *VSX1* gene, a 3′RACE experiment was performed on human adult retinal total RNA. Nested PCR using gene specific primers (Table 1, 3OA and 3IA) consistently yielded six polyadenylated products on gel electrophoresis (Figure 2). Duplication of the experiment using another set of primers (Table 1, 3OB and 3IB) produced similar results (data not shown). Sequence analysis of the 3′RACE product clones consistently revealed two novel downstream exons for *VSX1*, exon 6 and exon 7. Exon 6, 108 bp in length, is located at position 24993279–24993172 on genomic contig NT_011387. Exon 7 spans 535 bp of genomic DNA at position 24992119–24992654 (NT_011387). These two exons were mainly non-coding, not highly conserved and alternatively spliced in different *VSX1* transcripts. Including these novel exons, the *VSX1* gene now spans 10.65 kb of genomic DNA (former genomic size 6.7 kb).

**Figure 3:**
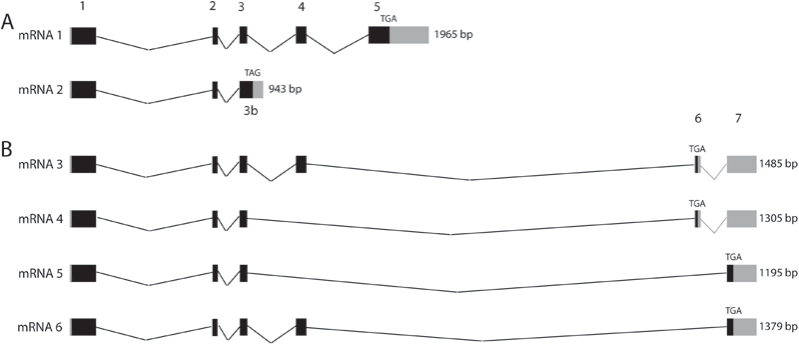
The mRNAs transcribed from the human *VSX1* gene. **A**: Previously described; **B**: novel transcript variants. Exons are numbered from 1 to 7. Black boxes indicate coding sequences; thin broken lines indicate spliced intronic sequences; gray boxes indicate untranslated regions. Numbers on the right show the length of each transcript variant as determined from the +1 position.

The full cDNA sequence for each of the transcript variants was deposited in GenBank (DQ854807 to DQ854812). Sequence of the 5′end of transcripts (5′UTR, exon 1 and 5′ end of exon 2) was obtained using previously reported *VSX1* sequence (GenBank NM_014588). Transcript variants 1 and 2 (GenBank NM_014588 and NM_199425) were previously described (Figure 3A) [32]. Transcripts 3 to 6 are novel (Figure 3B).

**Figure 4:**
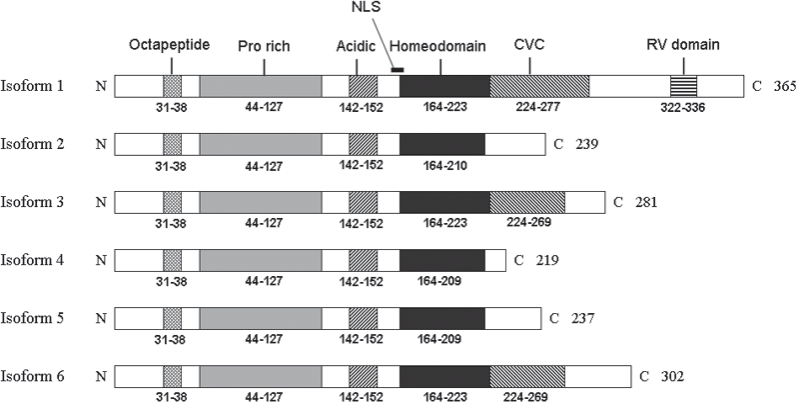
Schematic representation of VSX1 protein isoforms. Conserved domains of VSX1 and the numbered amino acid residues comprising each domain are indicated. The numbers on the right show total length of each isoform. N and C are amino and carboxyl termini of the isoforms. (NLS: nuclear localization signal; CVC: Chx10, Vsx1, ceh-10 domain; RV: RINX, Vsx1)

Transcript 1 has the longest ORF, encoding a 365 residue protein (38.43 kDa) containing homeodomain (HD), CVC (*Chx10*, *Vsx1* and *ceh-10*) and RV (*RINX* and *Vsx1*) domains and additional motifs previously described [32]. Other transcripts result in truncated protein products lacking RV domain, part or the whole CVC domain and sometimes the carboxyl end of the HD. The most carboxyl ends of these truncated polypeptides show no significant homology (Figure 4) to any known protein domains (Pfam software, provided in the public domain by the Sanger institute).

### Predicting *cis-*regulatory modules

To identify conserved non-genic sequences (CNGs) potentially related to *VSX1*, 110 kb of genomic sequence (50 kb upstream and downstream of the gene; chr20:24,950,120–25,060,767; UCSC hg17) was compared with orthologous sequences in 9 non-human vertebrates (dog, mouse, rat, opossum, chicken, frog, *Tetraodone*, zebrafish and *Fugu*). Publicly available, gapped and ungapped alignments available at Vista genome (provided in the public domain by the Genomics Division of Lawrence Berkeley National Laboratory) and ECR (provided in the public domain by the Dcode.org Comparative Genomics Center) browsers were used [33-35]. The analysis was performed under the established parameters for CNGs (70% identity in 100 bp window) [36,37]. These criteria revealed five upstream and one downstream evolutionary conserved regions (ECRs) with conservation between the human reference and multiple (at least three) other non-primate vertebrate lineages. Other ECRs in this region physically overlap exons of *VSX1* or the adjacent gene (*ACAS2L*). These six ECRs (5+1) are most likely non-coding as no corresponding spliced ESTs were detected (Table 2). This approach for identification of ECRs has been validated and established for identifying sequences with slower evolution than the average neutral rate [36].

**Table 2:** Amplicons containing ECRs in the vicinity of *VSX1*.

**ECR**	**Approximate size (bp)**	**ECR start position**	**ECR end position**
R1	256	25,012,020	25,012,275
R2	46	25,013,495	25,013,640
R3	2700	25,027,750	25,030,450
R4	410	25,045,600	25,046,010
R5	700	25,057,481	25,058,179
R6	2220 (two elements)	24,982,900	24,985,120

Sequence positions refer to the May 2004 release of human reference sequence (NCBI build 35).

Predicted ECRs consistently overlapped conserved transcription factor binding site (TFBS) clusters predicted by rVista 2.0 program (pre-computed annotation of ECR browser) [38]. Overlap with TFBSs supports the functional role of predicted ECRs. The conserved nature of these TFBS clusters suggests these could be *cis*-regulatory modules (CRMs) regulating *VSX1* expression.

### Mutation analysis of *VSX1* and related ECRs

Mutation analysis of *VSX1* in the family used for mapping PPCD1 locus (Family 1) as well as 2 other families suggested to be linked to PPCD1, included the five known exons, the two novel exons 6 and 7, and the end part of exon 3 specific to transcript variant 2 (exon 3b in Figure 3A) was carried out. No change was identified in selected individuals sequenced from these families.

We expanded our mutation analysis to include the ECRs around *VSX1*. Mutational analysis of amplicons containing the six identified ECRs (see above) failed to show mutations in Families 1 and 2. Three previously described SNPs were observed in Family 1. rs6050337 with a minor allele frequency of 0.267 (dbSNP) was identified in R4 (heterozygous). Minor alleles of two other SNPs (rs2224072 and rs4141461) were observed as homozygous in R3. None of the identified SNPs cosegregate with the disease status.

### Fine mapping of the PPCD1 critical region

No *VSX1* disease-causing mutations were found in the two PPCD families (Families 1 and 2) initially used for mapping the disease to chromosome 20. The 20 cM critical disease interval described by Héon et al. [6] also included *ID1*, a developmentally important dominant negative helix–loop–helix protein which did not show any mutation of the coding sequence in these families (unpublished data).

**Figure 5:**
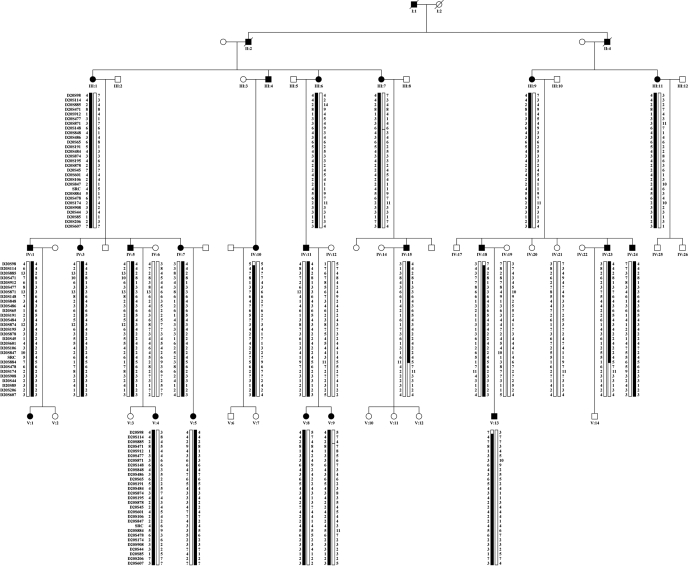
Pedigree structure of family 1 and its haplotype analysis at PPCD1 locus. This family was originally used to map PPCD to the chromosome 20 locus [6]. No *VSX1* mutation has been identified in this family. Marker names are shown on the right. Genotypes of each individual are shown as numbers underneath the individual's symbol. Dark bars represent the inferred disease haplotype with recombinations of parental chromosome shown.

**Figure 6:**
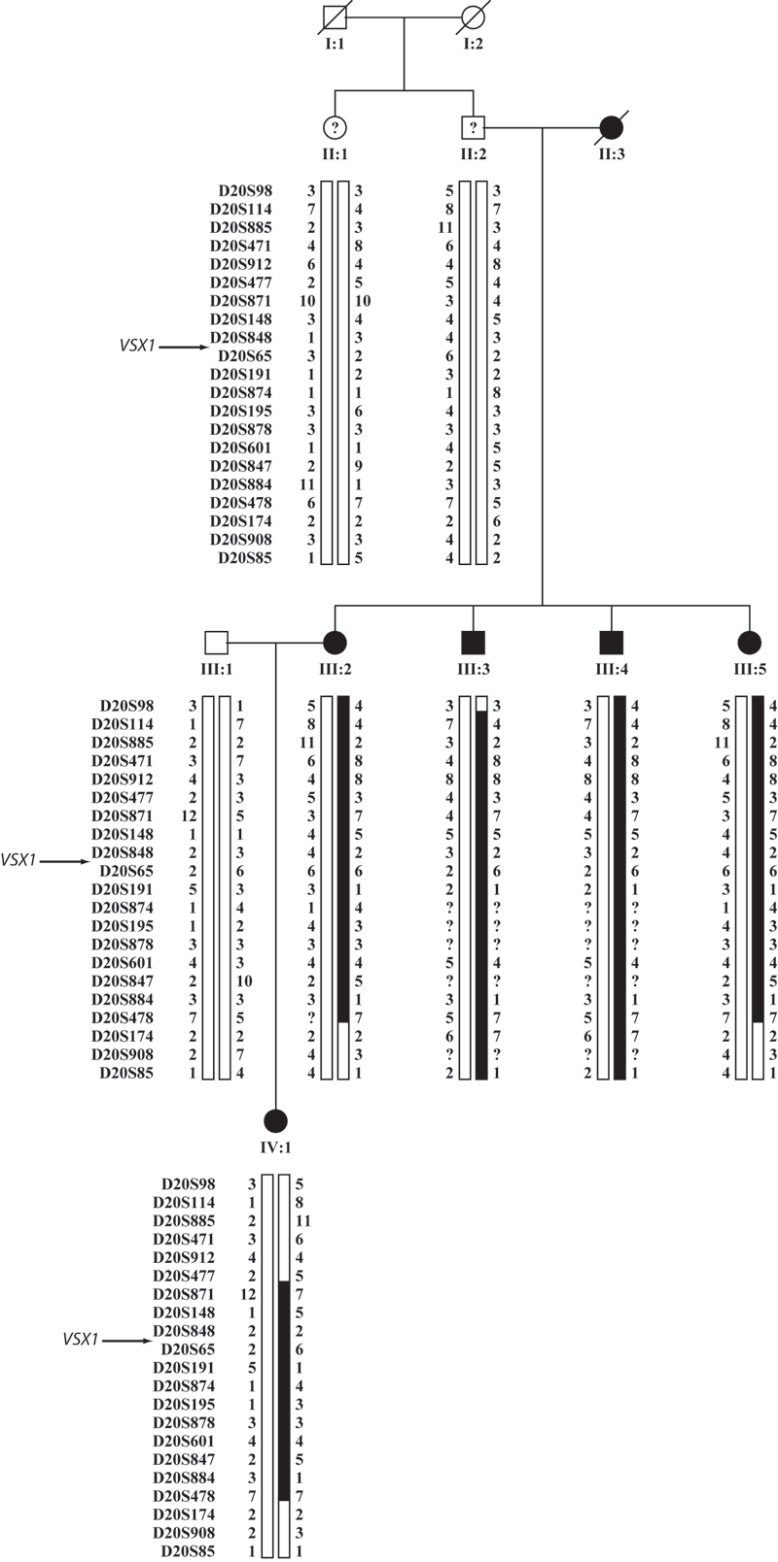
Haplotype analysis of family 3 at PPCD1 locus. The family has been fully described elsewhere [11] with mutations observed in *VSX1* coding sequence. Marker names are shown on the right. Genotypes of each individual are shown as numbers underneath the individual's symbol. Dark bars represent the inferred disease haplotype with recombinations of parental chromosome shown.

Therefore, mapping of the disease interval was refined for families 1 and 3 (Figure 5 and Figure 6). Family 2 (not shown) could not be used for narrowing down the interval due to the poor family structure (DNA available from 2 affected individuals). In Family 1 (Figure 5) recombination events narrowed down the critical interval to 16.44 cM (about 21.55 Mb). Further genotyping of family 3 (even though known to carry two *VSX1* changes; Figure 6) [11] narrowed the disease interval to 6.57 cM (about 14.8 Mb).

**Figure 7:**
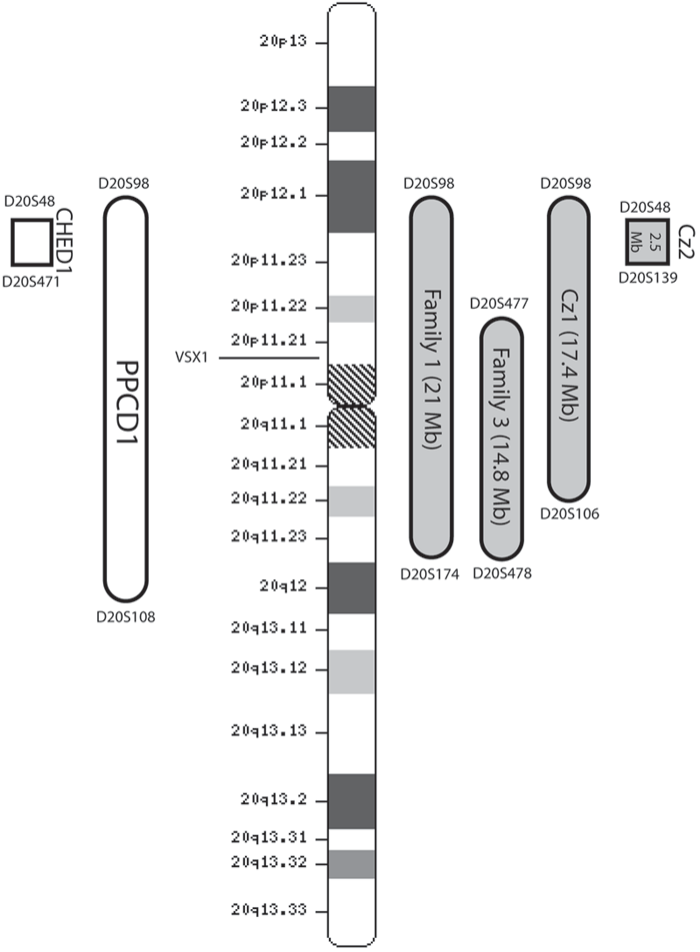
Ideogram of chromosome 20 showing PPCD1 minimal disease interval in different families. Bars on the left represent the disease intervals for PPCD1 and CHED1 as originally described [6,10]. Bars on the right show the refinements made in our study. Family 1 is the family used for mapping the locus (further refinement). Family 3 is the family described by Heon et al. [11] with a known *VSX1* mutation. Cz1 and Cz2 represent Czech families described by Gwilliam et al. [39].

There was no evidence of haplotype sharing between these families (data not shown). Figure 7 schematically summarizes the disease intervals for the PPCD1 families described to date including our recent data. These data suggest heterogeneity at PPCD1 locus by breaking it to two intervals. The disease interval for family 3, known to have a *VSX1* mutation, is definitely distinct from the CHED1-PPCD1 overlap interval described in Czech families (see discussion) [39].

### Mutation analysis of candidate genes from PPCD1-CHED1 interval

Genes in the PPCD1-CHED1 overlap interval were prioritized based on corneal expression and function. Available corneal EST and SAGE libraries were used as the initial evidence of corneal expression. Expression was further confirmed by RT–PCR on human corneal RNA [40-43]. The strongest three candidate genes included *RBBP9, ZNF133,* and *SLC24A3* (see discussion). We analyzed the coding sequence of these genes in individuals from both families (1 and 2) which did not reveal any mutation. Sequence analysis included the first seven exons of *SLC24A3* located in the PPCD1-CHED1 overlap interval.

## Discussion

Our study is the first documentation (and assessment) of *VSX1* expression in neonatal human cornea. This observation is significant since the role of *VSX1* as a candidate gene was questioned, partly due to its expression profile [44]. *VSX1* expression was proposed to be exclusive to a subset of inner nuclear layer cells of retina and absent from the adult human or developing mouse cornea [11,15,32]. The results from our study supported the findings of two recent murine studies [16,45] showing corneal expression of *VSX1*.The *VSX1* expression seems to be restricted to the perinatal period, which is consistent with the current hypothesis that PPCD-related abiotrophy begins during perinatal endothelial differentiation [46]. Electron microscopic studies on Descemet's membrane of PPCD cases, provides evidence that the pathology arises at gestation or shortly after birth [46,47].

We showed alternative splicing of *VSX1* and characterized six different transcript variants for this gene. Five of the six transcript variants identified encode truncated proteins. Isoforms 2, 4, and 6 are not expected to bind the DNA efficiently due to the lack of recognition helix of HD. Isoforms 3 and 6, which retain most of the DNA binding domains, should have significant DNA binding activity. Alternatively spliced transcripts encoding truncated isoforms have been described in several homeodomain genes [48-51]. The functional significance of *VSX1* truncated isoforms remains unclear. However, since all of these isoforms retain the proline-rich and acidic domains for transcriptional activation, they may modulate the transcriptional activity of the full-length protein by competing for co-activators or co-repressors. Another possibility is having independent developmental functions, through a mechanism other than DNA binding [50].

A thorough mutation analysis of novel *VSX1* exons and ECRs in the vicinity of the gene in the original PPCD1 family failed to reveal any disease causing mutation. It seems that *VSX1* plays a pathogenic role only in a subgroup of PPCD1 mapped families. *VSX1* changes observed in Family 3 were considered biologically significant (conservation and segregation with the disease status) [11] and convincing *VSX1* mutations have been reported in PPCD families from other groups [12-14]. Nonetheless, not identifying a disease-associated mutation in *VSX1's* genomic sequence, different transcript variants and regulatory elements, in some chromosome 20-linked PPCD families (including family 1) suggests that another gene may be involved [11,39].

Fine mapping of the disease interval in the family with an identified *VSX1* mutation (Family 3) showed that the smallest disease interval in this family is distinct from one of the reported Czech families [39], suggesting genetic heterogeneity of the original PPCD1 locus (Figure 7).

Autosomal dominant CHED (CHED1) and PPCD share several clinical, histopathologic, and immunohistochemical features, which suggest that these two conditions could be allelic variants. Overlap of PPCD1 and CHED1 loci (especially in the Cz2 family) is consistent with this concept of clinical heterogeneity [10,52]. This suggests that CHED1 may be a locus for a second PPCD gene on chromosome 20.

No other human ocular phenotype maps to the new PPCD1-CHED1 minimal disease interval (20p11.23-p12.1; OMIM, provided in the public domain by NCBI). Three ocular phenotypes (corneal disease-1, corneal disease 1- 2 Jackson, and blind-sterile) map to the syntenic region in the mouse genome on the distal part of chromosome 2 (Mouse Genome Informatics, provided in the public domain by the Jackson Laboratory). None of these conditions have direct relevance to corneal endothelium [53-55]. This interval contains more than 20 genes, some of which are interesting candidate genes based on expression and functional evidences.

We excluded the coding sequence of three additional candidate genes in our families. *RBBP9* (retinoblastoma binding protein 9), with high expression in corneal endothelium [40], has been shown to be important in the transformation process via its capacity to confer resistance to the growth-inhibitory effects of TGF-β1 [56]. PPCD occurs in consequence of metaplasia from a non-proliferating endothelium to an expanding epithelium. Interestingly, there is compelling evidence that the arrest of normal endothelium in the G1-phase of cell cycle is partly regulated by TGF-β (both β1 and β2) [57] which is known to be expressed by cultured human endothelial cells [58-60] and in aqueous humor [61-63]. *ZNF133,* a transcriptional repressor containing KRAB box and zinc finger domains [64,65], is a strong candidate gene with corneal expression [41,43,66,67]; since another zinc finger protein (*TCF8*) has been associated with PPCD [45]. *SLC24A3* (solute carrier family 24 member 3) shows corneal expression [68]. The interest in this K^+^-dependent Na^+^/Ca^2+^ exchangers is related to the recent association of another solute carrier (*SLC4A11*) with autosomal recessive CHED [69,70].

Endothelial corneal dystrophies are significant causes of visual impairment that would benefit from further molecular characterization. However, elucidation of the genetic basis of chromosome 20-related PPCD will require larger patient cohorts to fully evaluate the role of the regulatory elements and isoforms of *VSX1* as well as to validate the role of other mutational events or disease-associated genes.
